# Two-step artificial intelligence system for endoscopic gastric biopsy improves the diagnostic accuracy of pathologists

**DOI:** 10.3389/fonc.2022.1008537

**Published:** 2022-09-23

**Authors:** Yan Zhu, Wei Yuan, Chun-Mei Xie, Wei Xu, Jia-Ping Wang, Li Feng, Hui-Li Wu, Pin-Xiang Lu, Zi-Han Geng, Chuan-Feng Lv, Quan-Lin Li, Ying-Yong Hou, Wei-Feng Chen, Ping-Hong Zhou

**Affiliations:** ^1^ Endoscopy Center and Endoscopy Research Institute, Zhongshan Hospital, Fudan University, Shanghai, China; ^2^ Shanghai Collaborative Innovation Center of Endoscopy, Shanghai, China; ^3^ Department of Pathology, Zhongshan Hospital, Fudan University, Shanghai, China; ^4^ Ping An Healthcare Technology, Shanghai, China; ^5^ Department of Gastroenterology, Jiangyin Hospital Affiliated to Nanjing University of Chinese Medicine, Jiangsu, China; ^6^ Endoscopy Center, Central Hospital of Minhang District, Shanghai, China; ^7^ Department of Gastroenterology , Zhengzhou Central Hospital, Henan, China; ^8^ Endoscopy Center, Central Hospital of Xuhui District, Shanghai, China

**Keywords:** gastric cancer, endoscopy, artificial intelligence, pathology, gastric biopsy specimens

## Abstract

**Background:**

Endoscopic biopsy is the pivotal procedure for the diagnosis of gastric cancer. In this study, we applied whole-slide images (WSIs) of endoscopic gastric biopsy specimens to develop an endoscopic gastric biopsy assistant system (EGBAS).

**Methods:**

The EGBAS was trained using 2373 WSIs expertly annotated and internally validated on 245 WSIs. A large-scale, multicenter test dataset of 2003 WSIs was used to externally evaluate EGBAS. Eight pathologists were compared with the EGBAS using a man-machine comparison test dataset. The fully manual performance of the pathologists was also compared with semi-manual performance using EGBAS assistance.

**Results:**

The average area under the curve of the EGBAS was 0·979 (0·958-0·990). For the diagnosis of all four categories, the overall accuracy of EGBAS was 86·95%, which was significantly higher than pathologists (P< 0·05). The EGBAS achieved a higher κ score (0·880, very good κ) than junior and senior pathologists (0·641 ± 0·088 and 0·729 ± 0·056). With EGBAS assistance, the overall accuracy (four-tier classification) of the pathologists increased from 66·49 ± 7·73% to 73·83 ± 5·73% (P< 0·05). The length of time for pathologists to manually complete the dataset was 461·44 ± 117·96 minutes; this time was reduced to 305·71 ± 82·43 minutes with EGBAS assistance (P = 0·00).

**Conclusions:**

The EGBAS is a promising system for improving the diagnosis ability and reducing the workload of pathologists.

## Introduction

Gastric cancer is the fifth most common malignant cancer and the third leading cause of cancer-related mortality worldwide (1, 2). Gastroscopy is the pivotal procedure for the diagnosis, and especially the early detection, of the gastric cancer. The critical step of a gastroscopy examination is endoscopic biopsy when abnormal mucosa is detected. Additionally, endoscopic biopsy is the only way to make a definitive diagnosis of gastric mucosal diseases. In China, 22 million gastroscopy examinations are performed every year, creating potentially more than 50 million endoscopic biopsy specimens of gastric lesions (3). Although a rapid and precise diagnosis of gastric cancer is a necessary prerequisite for further treatment, the large number of endoscopic biopsy specimens combined with the current shortage of certified pathologists yield heavy workloads and affect diagnosis accuracy (4). According to the WHO 2019 classification system employed in China (5), visual inspection is not adequate to capture subtle changes in biopsy tissue because it may lead to different interpretations and introduce inter-and intra-observer variability.

Recently, artificial intelligence (AI) has been advancing in the field of pathological diagnosis to provide improved accuracy and save time (6, 7). Studies have applied conventional neural networks to help detect and diagnosis gastric cancer, achieving good results (8). However, an AI system based on the four-tier or five-tier classification system referred to in the latest WHO classification (5^th^ edition) dedicated to endoscopic gastric biopsy still does not exist. The pathological diagnosis of endoscopic gastric biopsy has many different characteristics compared with gastric cancer biopsies obtained from surgery, and they should be considered separately in actual diagnosis. The e-Pathologist image analysis software can be used to classify digitized histological images of gastric biopsy specimens into three categories: carcinoma or suspicion of carcinoma, adenoma or suspicion of a neoplastic lesion, and no malignancy which is the unique classification to the software. However, it has shown poor robustness in a clinical setting (9).

Moreover, pathologists combine all lesion areas in the entire biopsy rather than local patches (small images divided from biopsy) to make a final diagnosis. Deep convolutional neural networks (DCNNs) can only provide the results of each local patch due to hardware limitations. Some DCNN methods can determine the biopsy’s category based on the proportion of patch categories or feature fusion methods (10–12), which may affect the classification accuracy due to not considering the relationship among the patches. Graph convolutional networks (GCNs) can build relationships among irregular data and may have the potential to optimize this problem. GCN has been demonstrated to have good performance for classification in non-European spatial datasets (13–15).

In this study, we applied whole-slide images (WSI) of endoscopic gastric biopsy specimens (stained with H&E) expertly annotated to develop an endoscopic gastric biopsy assistant system (EGBAS) based on DCNNs and GCNs. The primary function of the EGBAS was to support pathologists in segmenting the important regions for diagnosis and help them screen out some negative cases rather than only provide a final diagnosis. We trained and externally validated the EGBAS on a large-scale, multicenter test dataset and compared its performance with the performance of pathologists with or without the assistance of the AI system.

## Materials and methods

### Dataset and annotation

The study was approved by the Institutional Review Board of Zhongshan Hospital, Fudan University (B2018-232R). All patients who underwent gastric biopsy at participating hospitals from June 2018 to June 2019 were retrospectively enrolled in the study. Biopsies were classified using the latest 5^th^ edition WHO classification of gastric tumor tissue. This classification includes four categories in order of increasing risk of malignancy: negative for dysplasia (NED), low-grade dysplasia (LGD), high-grade dysplasia (HGD), and intramucosal invasive neoplasia (IIN). Tissue classified as NED is characterized by normal gastric mucosa, gastric polyps, and inflammatory condition.

Gastric biopsy specimens were stained with H&E and mounted on glass slides by the participating hospital. Then, the slides were collected for the study and de−identified before WSI scanning. The slides used for training and validation of the EGBAS were obtained from Zhongshan Hospital (1023 for training, 106 for validation), Jiangyin Hospital Affiliated to Nanjing University of Chinese Medicine (120 for training, 16 for validation), and Central Hospital of Minhang District (1230 for training, 123 for validation). For the test dataset, slides were collected from multiple centers, including Zhongshan Hospital (ZS, 196 slides), Central Hospital of Xuhui District (XH, 195 slides), Central Hospital of Minhang District (MH, 940 slides), Zhengzhou Central Hospital (ZZ, 127 slides), and Jiangyin Hospital Affiliated to Nanjing University of Chinese Medicine (JY, 545 slides). A total of 2003 slides was reviewed by the EGBAS. **Table 1** presents the number of glass slides per dataset used in this study by WHO category. Considering human pathologists cannot read thousands of WSIs in one day, 429 slides (average number of endoscopic biopsies reviewed daily by pathologists at Zhongshan Hospital) were included in a man-machine comparison test dataset. All cases in this dataset were randomly selected from the test dataset and consisted of 104 NED, 83 LGD, 66 HGD, and 176 IIN slides.

**Table 1:** Number of whole-slide images of endoscopic biopsy specimens used in the training, validation, and test datasets.

Class	Training	Validation	Test
**NED**	885	80	1648
**LGD**	440	51	87
**HGD**	457	47	67
**IIN**	591	67	201
**Sum**	2373	245	2003

HGD, high-grade dysplasia; IIN, intramucosal invasive neoplasia; LGD, low-grade dysplasia; NED, negative for dysplasia.

The previously diagnosed glass slides of endoscopic biopsy specimens of gastric lesions were scanned using whole-slide imaging at ×40 magnification. The scanners include Aperio AT2 Scanscope Console SQS-1000, Jiangfeng KF-PRO-005, and Hamamatsu NanoZoomer S360. For the training dataset, pathologists identified the diagnostically relevant regions of interest (ROI) in the WSIs according to the 2019 WHO criteria. The annotation of ROI for model training was conducted using an NDP.view2 (version 2·7·39) annotation system.

The first step of the annotation procedure was labeling and verification. Three board-certified pathologists with at least 10 years of experience reviewed the WSIs and checked the final label with their pathological report. Next, a pathologist annotated the ROI on WSIs and then passed the annotation to another pathologist for review. The EGBAS was developed along with the annotation procedure, and the annotation provided by the system was sent to the pathologists to verify it was the important region for final diagnosis.

**Figure 1:**
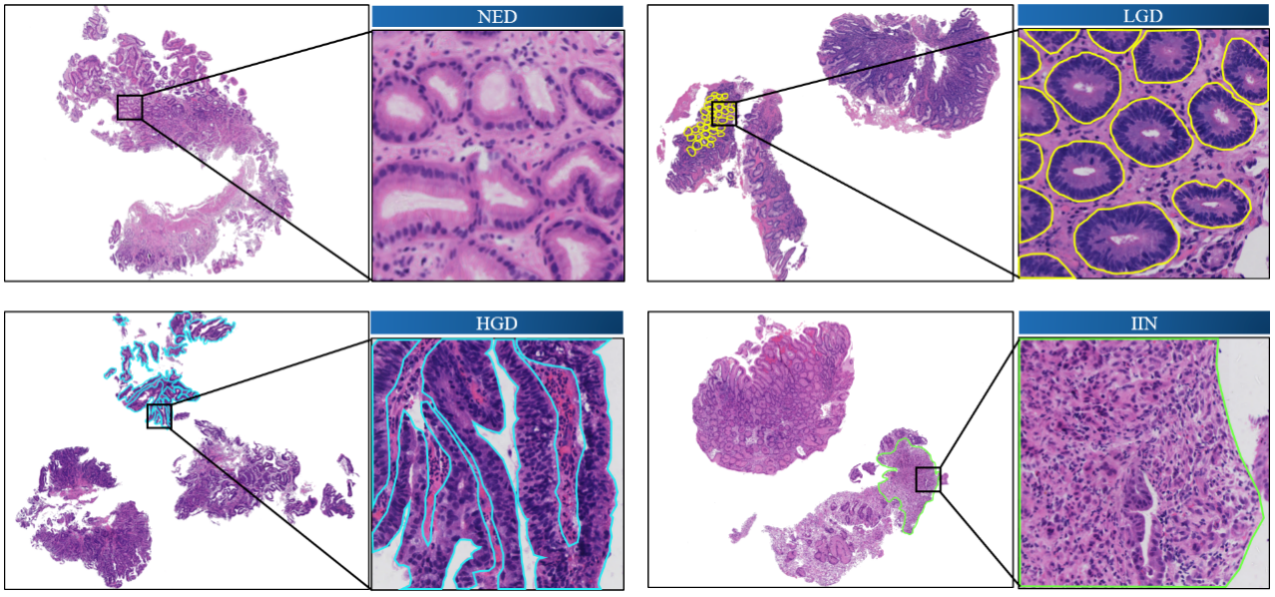
Annotated images of slide-mounted endoscopic biopsy specimens. Representative images of four categories (NED, LGD, HGD, and IIN) of gastric biopsy are presented. The abnormal regions distinct for each category are annotated with different colors. One local area is selected in each slide as a closeup image. HGD, high-grade dysplasia; IIN, intramucosal invasive neoplasia; LGD, low-grade dysplasia; NED, negative for dysplasia.

**Figure 1** shows the annotated endoscopic biopsy specimens, and different colors indicate the different categories. The undesired areas such as section identification, black border, or text annotations were removed before the annotation work.

### Image augmentation and stain normalization

Abundant training data are the key to promoting model performance, which prevents overfitting effectively. Thus, spatial [flipping, rotation, and random erasing (16)] and color (brightness, saturation, and contrast) augmentations, which could increase a dataset’s diversity, were applied in our method to enhance the model’s robustness.

The model in the study was trained on the slides from three centers (ZS, JY, and MH) and tested on slides from five different centers (ZS, XH, MH, ZZ, and JY). One major problem in multicenter testing was color variation caused by differences in chemical raw material, composition of staining protocols, or color response of digital scanners.

Stain normalization was employed to eliminate undesirable color variation in tissue area extracted by one automatic thresholding method (17). All tissue areas were linearly transformed into the same target mean and standard deviation in each channel, which were derived from the average of corresponding channel in the training dataset. Stain normalization was formulated as follows:


$$Z_{R}=\frac{X_{R}-\mu }{\sigma }\;\times \;\sigma_{R}+\mu_{R}$$

where *μ*_*R*_ and *σ*_*R*_ represents the target mean and standard deviation of channel R, *µ* and *σ* represents the mean and standard deviation calculated from current image of channel R, and *X*_*R*_ and *Z*_*R*_ are the original value and normalized value of pixels in the whole image. The same operations were used for the other two channels (G and B), and finally, the normalized three channels were merged into a new image.

### Algorithm development and training

**Figure 2:**
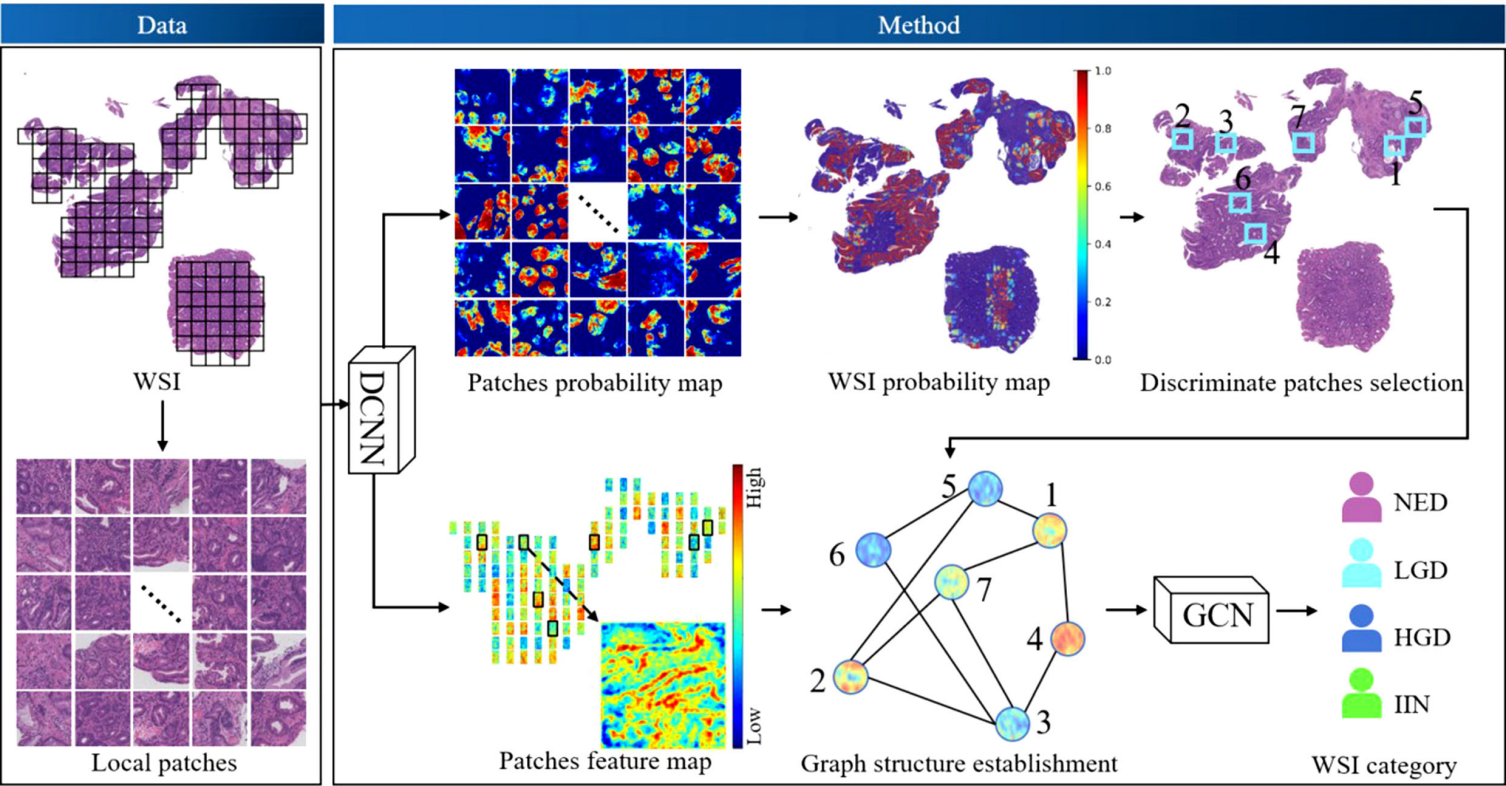
The workflow of the algorithm. Deep convolutional neural networks (DCNNs) and deep graph convolutional networks (GCNs) were used. DCNNs were selected to acquire the probability map of patches and extract the feature map of patches. Discriminative patches were selected with reference to the overall probability map of whole-slide images (WSIs), which was obtained by stitching all patch probability maps. Thus, the graph of WSIs was constructed based on discriminative patches and corresponding feature maps, and the graph was fed into the GCN model to obtain a final classification. HGD, high-grade dysplasia; IIN, intramucosal invasive neoplasia; LGD, low-grade dysplasia; NED, negative for dysplasia.

The workflow of our algorithm is depicted in **Figure 2**. Given the input WSIs, the probability map and feature map were first obtained through DCNNs, followed by discriminative patches selection. Then, graph structure was established based on discriminative patches and corresponding feature maps. Finally, the WSI category was acquired by GCNs. This section consists of three parts: DCNN design, GCN design, and implementation details and evaluation metrics.

#### DCNN design

In the architecture, the Vnet (18) model was selected to locate the ROI regions whose segmentation effectiveness has been widely demonstrated. The mean resolution of the images was approximately 50000 × 110000, which could not be directly used as an input of the model due to computation and storage limitation. A total of 2618 WSIs (2373 for training and 245 for validation) from ZS were divided into 104000 patches with a size of 512 × 512, including 72000 for training and 32000 for validation (training and validation patches came from different WSIs). We then trained the DCNN model based on these patches to predict the feature map and probability map of abnormal (including LGD, HGD and IIN) ratio. Therefore, the overall probability map of each WSI could be obtained by stitching the respective results of their patches.

#### GCN design

Each WSI was only placed in one category. Even though a scan may have shown several abnormal regions belonging to different categories, pathologists incorporated all lesion areas in the entire tissue rather than local patches to make a final diagnosis. To predict the gastric biopsy slide with human-like logic, our method used GCNs that could effectively build the relationship of irregular data structure in non-Euclidean space.

We defined the whole slide as a graph. The patches divided from the slide were set as nodes. The potential interaction between two patches were set as edges.

The process of constructing a graph consisted of discriminative node selection, node feature extraction, and edge generation. Discriminative nodes were selected through abnormal grading calculated from the probability map to reduce redundant information. Similar to the process of pathologists, only top *n* nodes in the abnormality ranking were used to build the graph, which could remove the interference from some pure negative regions. The DCNN was regarded as the feature extractor, and all selected discriminative patches in tissue area were extracted on behalf of the nodes. The edge between two nodes was based on Chebyshev distance of features: a smaller distance represented a closer potential interaction while a larger distance represented a farther interaction. We assigned an edge between node *m* and *n* only when the distance was smaller than threshold γ, as in the following formula:

$$E_{mn}=\{\begin{array}{c} 1\;if\;distancex{(}_{m},x_{n})<\gamma \\ 0\;if\;distancex{(}_{m},x_{n})\geq \gamma \end{array}$$

where  *x*_*m*_ and *x*_*n*_ represents the feature of nodes *m* and *n*, respectively; γ == 0 represents no edge in the graph; and γ == +∞ represents a fully connected graph. The WSIs in the training and validation datasets of the GCN were the same as those of the DCNN, with the only difference being that the GCN was based on WSI rather than local patches.

#### Implementation details and evaluation metrics

The proposed method was implemented using the deep learning framework Tensorflow v1·16 and Pycharm Community v2019·3·4. Adam was used as an optimizer with the initial learning rate of 1 × 10^−3^. The loss function of the DCNN and GCN were set to dice loss (18) and focal loss (19), respectively. To accelerate the training stage, multithreading was used on 4 Tesla V100 GPUs, and it took 32 hours for 200 epochs in total.

The receiver-operating curve was used as an evaluation metric in our method to reflect the classification performance. Each point on the curve corresponding to the sensitivity and specificity based on a threshold. A sample was set as positive only when the classification ratio obtained from the model was larger than the threshold. As the area under the curve (AUC) became larger, the classification accuracy improved.

### Output of the analysis result

Different modes of system’s output were developed for the actual usage demand. In order to facilitate the pathologists and relieve their heavy workload, two special modes, NED-screening and four-tier classification, were developed. Firstly, the NED-screening mode would be performed for detecting and distinguishing the malignant (LGD, HGD and IIN) and benign (NED) cases by 100% sensitivity. The negative probability (from 0·0 to 1·0) of each WSI would be predicted by EGBAS. The WSI would be regarded as benign with the probability greater than threshold *t*, otherwise be regarded as positive. The strategy of this mode was to remove part of the benign cases on the premise that the 100% negative predictive value should be guaranteed to avoid the miss diagnosis. Such a screening could alleviate the heavy workload of pathologists in practical diagnosis. Secondly, our system assisted the doctor to classify the remaining WSIs according to four-tier classification mode by giving the probabilities values of each category (*p*_*NEG*_ , *p*_*LGD*_ , *p*_*HGD*_ , *p*_*IIN*_ ), which were add up to 1 (*p*_*NED*_+*p*_*LGD*_+*p*_*HGD*_+*p*_*IIN*_=1 ). The model believed the WSI tend to be the category whose corresponding probability was highest, from which the WSI would be sorted into one of four types including NED, LGD, HGD and IIN.

### Algorithm assessment and comparison with pathologists

The class of each slide in the large-scale test dataset was determined by three leading pathology experts in digestive diseases. Slides with conflicting labeling were removed from the test dataset.

The man-machine comparison test dataset was fully automated read by the EGBAS in the four-tier classification mode and fully manually read by pathologists who participated in this research. Additionally, it was reviewed fully manually and semi-manually by pathologists without or with EGBAS assistance. The NED-screening mode was applied to alleviate the workload to the pathologists. All pathologists were invited *via* email or instant message.

To compare between fully automated and fully manual reading, pathologists read the WSIs in the test dataset independently and gave their final diagnosis (NED, LGD, HGD, and IIN). Then, diagnoses by the EGBAS and the pathologists were compared by elapsed time, sensitivity, specificity, and accuracy. Data are reported as the means ± standard deviation

The comparison study of fully manual and AI-assisted reading by the pathologists contained two parts. For the first reading, the pathologists read the WSIs in the man-machine comparison test dataset independently and gave their final diagnosis. In the second reading after a six-week washout period, the pathologists reviewed the same WSIs in the test dataset with the assistance of the AI system. The AI system was expected to help pathologists in a NED-screening mode. The pathologists read this part of the WSIs briefly and skip the diagnosis step to save time. Then, the system delineated the region critical of the remaining WSIs for the final diagnosis. The pathologists consulted this delineation before giving the final diagnosis. The system provided more than the classification result. The classification from the pathologists was compared with the label of the WSI in the test dataset. The performance of the pathologists with or without the assistance of the EGBAS was compared. To compare the results of the four-tier classification, agreement was assessed as the percent agreement and κ coefficients. κ coefficients ranged from 0·00 to 1·00 and were interpreted descriptively as follows: poor κ, less than 0·20; fair κ, 0·20-0·40; moderate κ, 0·40-0·60; good κ, 0·60-0·80; and very good κ, 0·80-1·00.

### Data availability

The dataset is governed by data usage policies specified by the data controller (Zhongshan Hospital, Fudan University). The WSIs in the test dataset in this study will be available for the testing of the AI system upon approval by the Institutional Review Boards of Zhongshan Hospital. Applications for data access can be directed to the corresponding authors. All authors had access to the study data and reviewed and approved the final manuscript.

## Results

### Performance of the EGBAS on a large-scale test dataset

**Figure 3:**
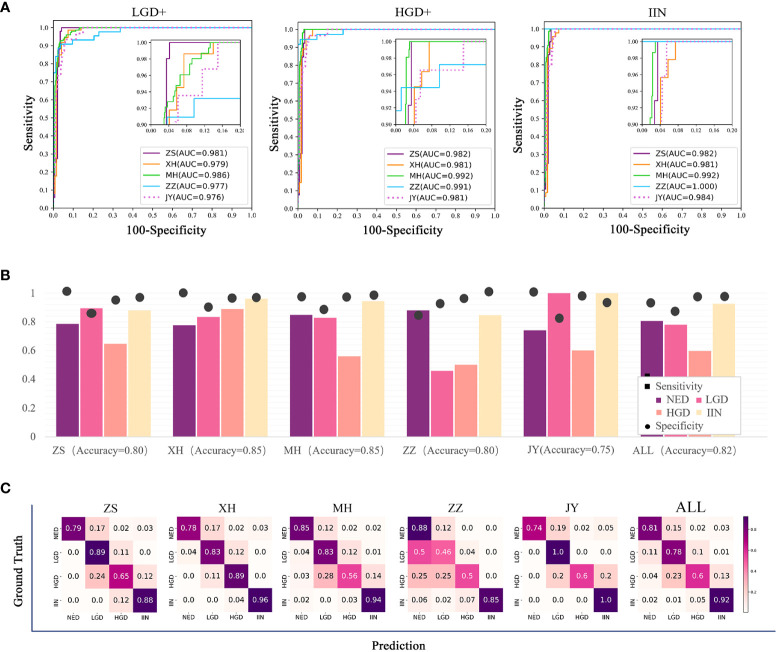
Results of the assessment of the endoscopic gastric biopsy assistant system on the large-scale test dataset. **(A)** The receiver-operating curve (ROC) of screening negative for dysplasia (NED) cases in four centers. The area under the curve (AUC) was 0·981, 0·979, 0·986, 0·977, and 0·976 in ZS, XH, MH, ZZ, and JY. The average AUC was 0·979 in the five centers. **(B)** Assessment of pathology slide classification. The accuracy, sensitivity, and specificity of detecting the four categories in all centers are presented. For NED, low-grade dysplasia (LGD), high-grade dysplasia (HGD), and intramucosal invasive neoplasia (IIN) categories, separate accuracy ranged from 0·750 to 0·850, sensitivity ranged from 0·632 to 0·926, and specificity ranged from 0·90 to 0·912. **(C)** Classification confusion matrix of the large-scale test dataset, which indicates the distribution of the predicted label on the true label. ZS, Zhongshan Hospital; XH, Central Hospital of Xuhui District; MH, Central Hospital of Minhang District; ZZ, Zhengzhou Central Hospital; JY, Jiangyin Hospital Affiliated to Nanjing University of Chinese Medicine.

All slides in test dataset (NED, 1648; LGD, 87; HGD, 67; and IIN, 201) were selected to evaluate the EGBAS’s performance of screening for NED. **Figure 3A** depicts three receiver-operating curves that represent the performance of screening for NED based on LGD+ (1648 NED, 87 LGD, 67 HGD, and 201 IIN), HGD+ (1648 NED, 67 HGD, and 201 IIN), and IIN (1648 NED and 201 IIN). The average AUC was 0·979, and AUC values of the five centers were all greater than 0·976.

**Table 2** shows that the specificity value on the condition of sensitivity is equal to 100% on screening for NED. The average accuracy, sensitivity, and specificity were 0·82, 0·79, and 0·88, and the standard deviation were 0·037, 0·151, and 0·078, respectively, across the centers, demonstrating a robust performance of the EGBAS on screening for NED.

**Table 2:** Sensitivity and specificity by category and center on screening for NED.

	ZS	XH	MH	ZZ	JY	ALL^*^
**Sensitivity**	100%	100%	100%	100%	100%	100%
**Specificity (LGD+)**	95%	84%	83%	66%	82%	83%
**Specificity (HGD+)**	96%	91%	94%	72%	82%	89%
**Specificity (IIN+)**	96%	92%	95%	100%	93%	94%

HGD, high-grade dysplasia; IIN, intramucosal invasive neoplasia; LGD, low-grade dysplasia; NED, negative for dysplasia.

^*^ALL was set up by merging other four test datasets.

The multiclassification performance of the EGBAS was also assessed based on the test dataset and four categories were existed to be classified. The mean accuracy was 0·80, 0·85, 0·85, 0·89, and 0·75 for ZS, XH, MH, ZZ, and JY, respectively (**Figure 3B**). The mean sensitivity was 0·81, 0·78, 0·60, and 0·92 for NED, LGD, HGD, and IIN (ranging from 0·74 to 0·88, from 0·46 to 1·0, from 0·50 to 0·89, and from 0·88 to 1·0; **Figure 3C**).

The average inference time of the EGBRAS for each WSI was 31·12 ± 7·54 seconds to obtain the final category prediction with one GPU (Tesla V100).

### The comparison of EGBAS with human diagnosis on the man-machine comparison test dataset

**Figure 4:**
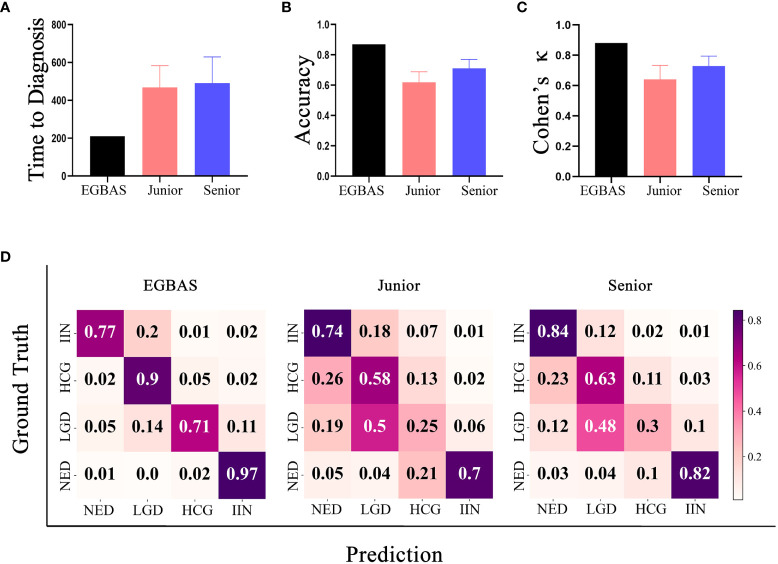
Results of the comparison between the endoscopic gastric biopsy assistant system (EGBAS) and pathologists using the man-machine comparison test dataset. **(A)** The length of time for the EGBAS and pathologists (junior and senior) to complete the dataset.**(B)** The overall accuracy of the EGBAS and pathologists (junior and senior). **(C)** The mean κ value of the EGBAS and pathologists (junior and senior). **(D)** A confusion matrix of the EGBAS, junior pathologists, and senior pathologists using the man-machine comparison test dataset.

Eight pathologists participated in our comparative study. They were all board-certified, achieved a master’s degree in pathology, and had more than 2 years of practical experience on the diagnosis of endoscopic gastric biopsy. Pathologists with at least four years of working experience were regarded as senior pathologists. All eight pathologists read the WSIs on a computer in the endoscopy center of Zhongshan Hospital under the supervision of our researcher. Additionally, this researcher measured and recorded the length of time needed for pathologists to read the scans and determine their final diagnosis. The average length of time to complete the dataset was 467·54 ± 22·50 for junior pathologists and 490·04 ± 90·96 minutes for senior pathologists. The EGBAS spent less time on diagnosis than both junior and senior pathologists (*P*< 0·05; **Figure 4A**). The EGBAS took 221 minutes to provide the final diagnosis and delineate the region critical to the final diagnosis with one GPU (Tesla V100).

Concerning the diagnosis of all categories, the overall accuracy of the EGBAS was 86·95%. By contrast, the eight pathologists achieved an overall accuracy of 66·49 ± 7·73%. The accuracy of junior and senior pathologists was 61·95 ± 9·09% and 71·04 ± 4·59%, respectively. Therefore, the EGBAS achieved a higher accuracy than the pathologists (*P*< 0·05). No significant difference was found between the accuracy for the two groups of pathologists (*P* = 0·09; **Figure 4B**).

The AI system achieved a higher κ score of 0·880 (very good κ) than junior and senior pathologists (0·641 ± 0·088 and 0·729 ± 0·056; **Figure 4C**); however, this difference was not significant. **Figure 4D** presents the confusion matrices of the four-tier classification of the EGBAS, junior pathologists, and senior pathologists on the man-machine comparison test dataset. The deeper color represents the better performance on the specific classification. Overall, the EGBAS showed better prediction on the classification of LGD, HGD, and IIN than the pathologists.

### The comparison between pathologists with or without the assistance of the EGBAS

The length of time for the pathologists to complete the first reading was 461·44 ± 117·96 minutes. With the assistance of the EGBAS, this time was reduced to 305·71 ± 82·43 minutes for the second reading (*P* = 0·00). Among all the time saved, the pathologists saved 100·63 ± 46·94 minutes due to spending less time on the NED cases diagnosed by the EGBAS.

Concerning the diagnosis of all four categories, the eight pathologists achieved an overall accuracy of 66·49 ± 7·73% for the first reading; this accuracy increased to 73·83 ± 5·73% for the second reading (*P*< 0·05).

**Figure 5:**
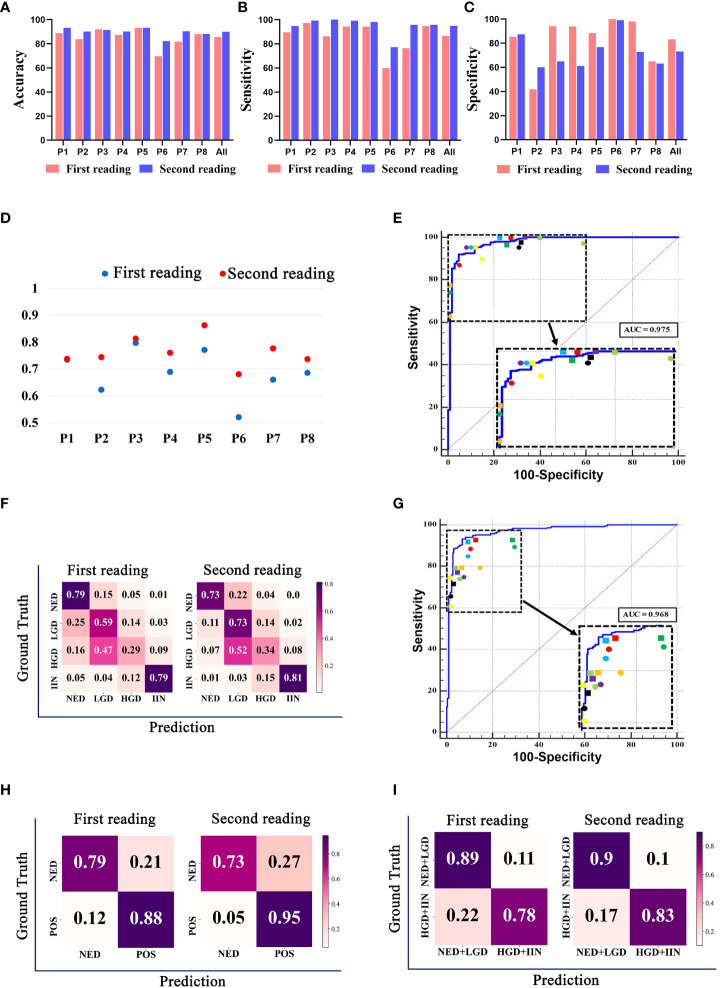
The classification results of pathologists with or without endoscopic gastric biopsy assistant system (EGBAS) assistance. **(A-C)** The accuracy, sensitivity and specificity of different categories for the first reading (manual reading) and second reading (AI-assisted reading) in a two-tier classification (NED vs. LGD, HGD, and IIN). P1-P8 represent 8 pathologists. **(D)** Cohen’s κ for each pathologist on four-tier classification. Each κ value is the average pairwise κ for each pathologist in two tests. **(E)** Results of a two-tier classification (NED vs. LGD, HGD, and IIN) by pathologists. The receiver-operating curve (ROC) was acquired from the EGBAS, and the points represent the performance of eight pathologists. The dots represent the first reading, and the diamonds represent the second reading. Different colors denote the performance of different pathologists. **(F)** A confusion matrix of the four-tier classification on the test dataset for the first reading and second reading. **(G)** Results of another two-tier classification (NED and LGD vs. HGD and IIN) by pathologists. The ROC was acquired from the EGBAS, and the points represent the performance of eight pathologists. The dots represent the first reading, and the diamonds represent the second reading. Different colors denote the performance of different pathologists. **(H)** A confusion matrix of the two-tier classification (NED vs LGD, HGD and IIN) on the test dataset for the first and second reading. POS, positive (LGD, HGD and IIN). **(I)** A confusion matrix of the two-tier classification (NED and LGD vs. HGD and IIN) on the test dataset for the first and second readings.

We compared the performance of the pathologists with or without the assistance of the EGBAS in a two-tier classification (NED vs. LGD, HGD, and IIN). The average accuracy, sensitivity, and specificity of the eight pathologists for the first reading (manual reading) were 85·51 ± 7·40%, 86·58 ± 12·71%, and 83·33% ± 20·05% (**Figures 5A–C**). For the second reading, the accuracy and sensitivity increased to 89·89 ± 3·51% and 94·99 ± 7·40% (*P* = 0·032 and 0·014); the specificity was 73·18 ± 13·96% *(P=* 0·15). For the first reading without AI assistance, the agreement with the reference standard (measured by the median quadratically weighted Cohen’s κ) for the pathologists was 0·685. For the second reading assisted by the EGBAS, the median κ of the panel increased to 0·764 (good κ), indicating a significant increase in performance (*P*< 0·05) (**Figure 5D**). The EGBAS achieved 93·01% accuracy in distinguishing between NED and other positive diagnosis (LGD, HGD, and IIN) with a mean AUC value of 0·975 ± 0·01 (0·955-0·988; **Figure 5E**). The confusion matrix of the pathologists for the first reading and second reading (**Figure 5F**) showed less NED specimens were misdiagnosed as LGD, HGD, or IIN with the assistance of the EGBAS.

Considering the different treatments for HGD and IIN, we also compared the performance of the pathologists in another two-tier classification (NED and LGD vs. HGD and IIN). The EGBAS achieved 93·01% accuracy in distinguishing between NED and other positive diagnoses (LGD, HGD, and IIN) with a mean AUC value of 0·968 ± 0·010 (0·946-0·982, **Figure 5G**). The average accuracy, sensitivity, and specificity of the eight pathologists for the first reading (manual reading) were 82·69 ± 3·57%, 77·48 ± 10·59%, and 89·13 ± 9·63%. For the second reading, the accuracy increased to 86·04 ± 2·45% (*P* = 0·046) while the sensitivity and specificity were 82·96 ± 8·14% and 89·84 ± 9 78% (*P* = 0·27 and 0·88). Two different types of two-tier classification confusion matrix were shown in **Figures 5H** and **I**. **Figure 5H** (NED vs. LGD, HGD, and IIN) shown pathologists achieved better performance in detecting LGD, HGD, or IIN with the assistance of AI system, however, lower accuracy in NED. Another two-tier classification shown in **Figure 5I** (NED and LGD vs. HGD and IIN) revealed that more lesions that required endoscopic or surgical intervention were detected and less lesions were misdiagnosed as HGD or IIN with the assistance of the EGBAS.

## Discussion

Gastric cancer is a frequent diagnosis and a leading cause of cancer death in China, accounting for 679,000 new cases and 498,000 deaths in 2015 (20). Endoscopic examinations with biopsy serve a primary role in the diagnosis and surveillance of patients with gastric precancerous conditions. Endoscopists with higher endoscopic biopsy rates have higher rates of detecting gastric premalignant conditions and lower rates of missing gastric cancer (21). However, a higher endoscopic biopsy rate is also associated with a higher negative biopsy rate and a heavier burden for the pathologists. As a solution, AI can help ease this burden for pathologists.

Considerable debate exists about the best approach for AI systems to help pathologists classify tissues. Some deep learning algorithms have been applied in histopathologic classification and have achieved a better diagnostic performance than pathologists in liver cancer (22), breast cancer (23), melanoma (24), and prostate cancer biopsy (25, 26). Previous results have showed that deep learning can detect a huge amount of data critical for the final diagnosis that are not accessible to visual inspection. With the increasing use of WSI for pathological diagnosis, more advantages have been discovered compared with the use of glass slides (27). The endoscopic biopsy specimens of gastric lesions are small, and one slide is enough for a case. Compared with specimen obtained from other ways, we found that the endoscopic biopsy specimen is perfectly suited to the use of WSI for primary diagnosis. According to research by Yoshida et al., the e-Pathologist software yield unsatisfactory results for three-tier classification of gastric biopsy WSI (28). The overall concordance rate was 55·6% and the κ coefficient was 0·28 (fair agreement). Song et al. detected the gastric cancer region in WSIs using DCNN and obtained slide-level prediction by averaging the top 1000 probabilities (29). The tissue examined in their study included both endoscopic biopsy and surgical specimens. The model achieved a sensitivity of near 100% and an average specificity of 80·6% on a real-world test dataset and performed robustly with WSIs from other medical centers. This research showed the feasibility and benefits of using AI systems in routine practice scenarios. Ziba et al. (30) built a deep learning–based system to classify breast histopathological images in two steps. First, the model was trained to classify the patches, and second, a decision tree combined the patches to obtain a final diagnosis. Additionally, Wang et al. (31) obtained whole-slide gastric image classification relying on two stages: first, selecting patches, and second, utilizing these patches to diagnose disease based on deep learning.

The application of a two-stage algorithm can effectively combine local and global information. However, these models did not consider the potential interactions among local patches, which could greatly influence the diagnosis of pathologists. Considering the evolution and spread of the disease, pathologists always incorporate all suspected lesion areas in the entire tissue and develop a pattern of relationships among them to make a final decision. To predict the gastric biopsy slide with human-like logic, our method used a GCN that could build the relationship of features extracted from discriminate patches as a graph in non-Euclidean space and yield a slide-level category by comprehensive training. With the assistance of the EGBAS, the overall time for pathologists to diagnose was only 0·662 times as much as the fully manual review, and the accuracy rate increased from 66% to 73%. As far as we know, this is the first research on an AI system that aimed to subdivide endoscopic biopsies into four categories (LGD, HGD, IIN, and NED) and was verified as helpful to pathologists’ diagnosis.

Referring to the existing research, we identified some practices that need to be emphasized in an AI system before it could be established as an ideal AI system for clinical application. First, the deep learning algorithm should be established based on large number of specimen slides collected from multiple centers, and the WSIs should be scanned using different brands of scanner devices to improve generalization. Additionally, algorithms should be developed to normalize WSIs collected from different centers. Second, the system should focus on assisting pathologists to improve their diagnostic accuracy rather than providing a diagnostic result. A semi-automated approach will not reduce workload as much as a fully automated approach, but its performance might improve the diagnostic ability of pathologists. Indeed, the pathologist is the one who should be responsible for the diagnosis rather than the AI system. Therefore, reducing pathologists’ workload and improving their accuracy should be the primary goal for the AI system. Third, the algorithm should be trained to simulate real-world diagnosis and performed in a multi-category, complex working pattern. In the real world, the pathologists have to face a broad spectrum of various diseases and even some orphan diseases to obtain the final diagnosis. The binary classification does not fit the actual work. In our research, we divided the complex WHO 2019 classification of endoscopic gastric biopsy into a four-tier selection. Fourth, the test dataset for verifying the performance of the AI system should contain WSIs collected from multiple centers to ensure the stability of the system. These practices presented a huge challenge for us and we made great efforts to meet these practices. They were also considered to be the general rules and guidelines for our study design of the EGBAS.

Considering the large number of patients that require endoscopic examination for screening, the cost-effectiveness should be taken into account to ease the heavy medical burden. To some extent, our system might be the perfect solution for this dilemma because it can screen NED specimens with negative predictive value, which can alleviate pathologists’ workload and speed up the diagnosis. Its two-tier classification can detect and distinguish malignant (LGD, HGD, and IIN) and benign (NED) cases with 100% sensitivity before providing the final diagnosis. The high sensitivity ensures the system will avoid missing the diagnosis of lesions that should be treated with endoscopic or surgical intervention. Thus, pathologists will be able to pay less attention to these benign cases and can focus on the malignant cases. In our research, pathologists saved approximately 156 minutes with the assistance of the EGBAS, and of this time, approximately 100 minutes were saved due to spending less time on the NED cases diagnosed by the EGBAS. This remarkable result demonstrated that pathologists can save valuable time with the assistance of the EGBAS. Thus, endoscopists can raise their endoscopic biopsy rate and also perhaps decrease the rate of false negatives (21).

Our research proved that the EGBAS can help pathologists to not only save time but also improve the accuracy of their diagnosis. The overall accuracy was increased to 73·83% in the four-tier classification, and the κ score was raised to 0·764 with the assistance of the EGBAS. Additionally, the accuracy significantly increased to 89·89% (NED vs. LGD, HGD, and IIN) and 86·04% (NED and LGD vs. HGD and IIN) in two different two-tier classifications. The results of our research demonstrated that the system can focus on delineating the important regions related to final diagnosis for pathologists to improve the accuracy of their diagnosis. The delineation on WSIs could help them highlight the important regions rather than interfere with their diagnosis. The classification of the biopsy tissue is always a tough task due to the existence of various histological conditions such as necrosis, hyperplasia, inflammation, and structural abnormalities in gastric biopsy tissues. Additionally, this task is made even more difficult by the process to distinguish benign lesions from malignant tumors. Sometimes, LGD cases are misdiagnosed as NED cases, causing patients to obtain a wrong follow-up protocol. The primary reason for this misdiagnosis is that the pathologist neglected some abnormal regions, which can be small in the WSI. With the help of the EGBAS, abnormal regions are delineated from benign tissue so that small regions also can attract the attention of pathologists, greatly reducing the false negative rate. Comparing with the system that only provides a classification result, our system can better fit in a wide range of regions who adopt different classification criteria (32, 33).

There are several limitations in this study. First, the EGBAS did not quantitatively analyze gastric atrophy and inflammation in H&E-stained WSIs; in the future, we will add this to the system to provide more information to guide further treatment. Second, gastric neuroendocrine tumor and gastric lymphoma were not considered as a diagnosis because immunohistochemical staining is needed to diagnose them. We aim to train the model to detect more diseases in the future. Third, we did not include an unclassified label in this research. In a pathologist’s daily routine, some cases will be diagnosed as unable to be classified due to the low quality of the slides or improper biopsy by endoscopists.

In summary, our study showed promising results that the EGBAS can improve the diagnosis ability of pathologists and reduce their workload. Additionally, the system can reduce intra-observer variability and help pathologists focus on the regions important to the final diagnosis. This is the first research about using an AI system for four-tier classification of endoscopic gastric biopsy, and further improvements will be made to apply the EGBAS in routine practice.

## Data availability statement

The raw data supporting the conclusions of this article will be made available by the authors, without undue reservation.

## Ethics statement

The study was approved by the Institutional Review Board of Zhongshan Hospital, Fudan University (B2018-232R). The patients/participants provided their written informed consent to participate in this study. Written informed consent was obtained from the individual(s) for the publication of any potentially identifiable images or data included in this article.

## Author contributions

P-HZ and Q-LL designed and supervised the study; YZ, WY, W-FC, and Z-HG participated in the design of the study and performed all data collection and analysis; C-MX was responsible for the construction of the AI system; J-PW, and C-FL provided the technical support of the algorithm; WX, LF, H-LW, P-XL, and Y-YH reviewed all the WSIs enrolled in the research and provided professional advice. All authors have read and approved the manuscript.

## Funding

This study was supported by grants from the National Key R&D Program of China (2019YFC1315800), National Natural Science Foundation of China (82170555, 82203193), Shanghai Rising-Star Program (19QA1401900), Major Project of Shanghai Municipal Science and Technology Committee (19441905200), and Shanghai Sailing Programs of Shanghai Municipal Science and Technology Committee (19YF1406400).

## Acknowledgments

We thank Pu Wang, Professor of Sichuan Academy of Medical Sciences & Sichuan Provincial People’s Hospital for his help in interpreting the significance of the results of this study. We thank Professor Chen Xu and Ya-Lan Liu from Zhongshan Hospital for their professional advice on the pathological analysis and the golden standard of the WSIs enrolled in the study. We thank Professor Wei Zhang from Fudan University and professor Xiao-Hong Zhang from Central Hospital of Minhang District for their suggestions about the methods of man-machine comparison test. We thank Jia-Yan Wang, Chang Xiao, Qiu-Cheng Wang, Feng-Yi Li, Pei-Yao Fu, Dan-Feng Zhang, Yang Nan, Guo-Tong Xie and Kun-Kun Li for their kindness support on this research.

## Conflict of interest

The authors declare that the research was conducted in the absence of any commercial or financial relationships that could be construed as a potential conflict of interest.

## Publisher’s note

All claims expressed in this article are solely those of the authors and do not necessarily represent those of their affiliated organizations, or those of the publisher, the editors and the reviewers. Any product that may be evaluated in this article, or claim that may be made by its manufacturer, is not guaranteed or endorsed by the publisher.

## Abbreviations

AI, artificial intelligence; DCNNs, deep convolutional neural networks; GCNs, graph convolutional networks; WSI, whole-slide images; EGBAS, endoscopic gastric biopsy assistant system; NED, negative for dysplasia; LGD, low-grade dysplasia; HGD, high-grade dysplasia; IIN, intramucosal invasive neoplasia; ROI, regions of interest.
